# Supplementary material for: Modeling glioblastoma heterogeneity as a dynamic network of cell states
Source: Mol Syst Biol. 2021 Sep 16;17(9):e10105. doi: 10.15252/msb.202010105 (PMC8444284; doi:10.15252/msb.202010105)
Supplement: Supplementary file 6 — Source Data for Figure 5 [file MSB-17-e10105-s004.zip › Figure5A_sourcedata/GSEA_3071/nosigdb_stateI.GseaPreranked.1622795145664/DOROSZKO_U3054MG_CLUSTER_2.html]

Details for gene set DOROSZKO\_U3054MG\_CLUSTER\_2[GSEA]

|  || Dataset | state13071 |
| Phenotype | NoPhenotypeAvailable |
| Upregulated in class | na\_pos |
| GeneSet | DOROSZKO\_U3054MG\_CLUSTER\_2 |
| Enrichment Score (ES) | 0.5434692 |
| Normalized Enrichment Score (NES) | 3.703183 |
| Nominal p-value | 0.0 |
| FDR q-value | 0.0 |
| FWER p-Value | 0.0 |
Table: GSEA Results Summary

  

Fig 1: Enrichment plot: DOROSZKO\_U3054MG\_CLUSTER\_2      
 Profile of the Running ES Score & Positions of GeneSet Members on the Rank Ordered List

  

| PROBE | GENE SYMBOL | GENE\_TITLE | RANK IN GENE LIST | RANK METRIC SCORE | RUNNING ES | CORE ENRICHMENT || 1 | COL3A1 |  |  | 3 | 2.646 | 0.0135 | Yes |
| 2 | LUM |  |  | 4 | 2.203 | 0.0255 | Yes |
| 3 | DCN |  |  | 5 | 2.010 | 0.0364 | Yes |
| 4 | COL1A1 |  |  | 6 | 2.008 | 0.0473 | Yes |
| 5 | PTCH1 |  |  | 7 | 1.951 | 0.0580 | Yes |
| 6 | PDGFRB |  |  | 9 | 1.893 | 0.0680 | Yes |
| 7 | FN1 |  |  | 11 | 1.769 | 0.0773 | Yes |
| 8 | COL6A3 |  |  | 13 | 1.717 | 0.0863 | Yes |
| 9 | COL5A1 |  |  | 14 | 1.706 | 0.0956 | Yes |
| 10 | PCOLCE |  |  | 15 | 1.696 | 0.1048 | Yes |
| 11 | CARMN |  |  | 19 | 1.663 | 0.1130 | Yes |
| 12 | NUPR1 |  |  | 21 | 1.585 | 0.1213 | Yes |
| 13 | RARRES3 |  |  | 22 | 1.575 | 0.1299 | Yes |
| 14 | SSPN |  |  | 23 | 1.574 | 0.1384 | Yes |
| 15 | BGN |  |  | 24 | 1.568 | 0.1470 | Yes |
| 16 | COL4A1 |  |  | 28 | 1.524 | 0.1543 | Yes |
| 17 | CFH |  |  | 29 | 1.523 | 0.1626 | Yes |
| 18 | POSTN |  |  | 32 | 1.494 | 0.1702 | Yes |
| 19 | COL11A1 |  |  | 37 | 1.468 | 0.1769 | Yes |
| 20 | BICC1 |  |  | 38 | 1.463 | 0.1849 | Yes |
| 21 | MFAP2 |  |  | 44 | 1.395 | 0.1910 | Yes |
| 22 | IGFBP7 |  |  | 45 | 1.387 | 0.1985 | Yes |
| 23 | SCN9A |  |  | 46 | 1.381 | 0.2060 | Yes |
| 24 | COL5A2 |  |  | 48 | 1.373 | 0.2132 | Yes |
| 25 | CDH11 |  |  | 59 | 1.296 | 0.2172 | Yes |
| 26 | PLXDC2 |  |  | 61 | 1.265 | 0.2238 | Yes |
| 27 | COLEC12 |  |  | 63 | 1.241 | 0.2302 | Yes |
| 28 | TFPI |  |  | 64 | 1.241 | 0.2370 | Yes |
| 29 | BHLHE41 |  |  | 69 | 1.218 | 0.2424 | Yes |
| 30 | SERPINF1 |  |  | 71 | 1.211 | 0.2487 | Yes |
| 31 | COL4A2 |  |  | 73 | 1.203 | 0.2549 | Yes |
| 32 | OMD |  |  | 75 | 1.196 | 0.2611 | Yes |
| 33 | APOD |  |  | 76 | 1.195 | 0.2676 | Yes |
| 34 | PPIC |  |  | 77 | 1.195 | 0.2742 | Yes |
| 35 | SNED1 |  |  | 78 | 1.191 | 0.2806 | Yes |
| 36 | CD248 |  |  | 79 | 1.190 | 0.2871 | Yes |
| 37 | NRP1 |  |  | 81 | 1.180 | 0.2932 | Yes |
| 38 | LRP1 |  |  | 84 | 1.160 | 0.2989 | Yes |
| 39 | ID3 |  |  | 86 | 1.152 | 0.3049 | Yes |
| 40 | FLRT2 |  |  | 89 | 1.131 | 0.3104 | Yes |
| 41 | SRPX2 |  |  | 90 | 1.123 | 0.3166 | Yes |
| 42 | COL12A1 |  |  | 91 | 1.117 | 0.3226 | Yes |
| 43 | ASPN |  |  | 92 | 1.117 | 0.3287 | Yes |
| 44 | ATP13A2 |  |  | 96 | 1.100 | 0.3338 | Yes |
| 45 | NOTCH3 |  |  | 98 | 1.096 | 0.3394 | Yes |
| 46 | MXRA8 |  |  | 100 | 1.090 | 0.3451 | Yes |
| 47 | ARL4C |  |  | 108 | 1.062 | 0.3487 | Yes |
| 48 | SLC2A3 |  |  | 109 | 1.060 | 0.3545 | Yes |
| 49 | COL8A1 |  |  | 111 | 1.056 | 0.3599 | Yes |
| 50 | S1PR3 |  |  | 114 | 1.043 | 0.3650 | Yes |
| 51 | RASL12 |  |  | 115 | 1.037 | 0.3707 | Yes |
| 52 | EMP1 |  |  | 122 | 1.012 | 0.3743 | Yes |
| 53 | OLFML3 |  |  | 125 | 0.971 | 0.3790 | Yes |
| 54 | GPX8 |  |  | 133 | 0.959 | 0.3821 | Yes |
| 55 | KDELR3 |  |  | 136 | 0.951 | 0.3867 | Yes |
| 56 | FBN1 |  |  | 138 | 0.944 | 0.3915 | Yes |
| 57 | INHBA |  |  | 140 | 0.941 | 0.3963 | Yes |
| 58 | SNAI2 |  |  | 142 | 0.940 | 0.4011 | Yes |
| 59 | LSP1 |  |  | 143 | 0.930 | 0.4062 | Yes |
| 60 | SERTAD4 |  |  | 149 | 0.915 | 0.4096 | Yes |
| 61 | CTGF |  |  | 152 | 0.913 | 0.4140 | Yes |
| 62 | SYT1 |  |  | 155 | 0.906 | 0.4183 | Yes |
| 63 | AHNAK |  |  | 163 | 0.887 | 0.4210 | Yes |
| 64 | TMEM100 |  |  | 170 | 0.872 | 0.4239 | Yes |
| 65 | PCDH18 |  |  | 171 | 0.870 | 0.4287 | Yes |
| 66 | FAM114A1 |  |  | 174 | 0.864 | 0.4328 | Yes |
| 67 | EGFR |  |  | 175 | 0.864 | 0.4375 | Yes |
| 68 | GLT8D2 |  |  | 181 | 0.856 | 0.4406 | Yes |
| 69 | ISLR |  |  | 183 | 0.853 | 0.4449 | Yes |
| 70 | DNM1 |  |  | 184 | 0.853 | 0.4496 | Yes |
| 71 | P3H1 |  |  | 186 | 0.849 | 0.4539 | Yes |
| 72 | COL21A1 |  |  | 191 | 0.847 | 0.4573 | Yes |
| 73 | ECE1 |  |  | 195 | 0.839 | 0.4609 | Yes |
| 74 | VCAN |  |  | 197 | 0.833 | 0.4652 | Yes |
| 75 | EMILIN1 |  |  | 207 | 0.812 | 0.4669 | Yes |
| 76 | EBF1 |  |  | 221 | 0.783 | 0.4672 | Yes |
| 77 | OLFML2B |  |  | 233 | 0.763 | 0.4680 | Yes |
| 78 | SEC24D |  |  | 235 | 0.761 | 0.4718 | Yes |
| 79 | MSRB3 |  |  | 242 | 0.743 | 0.4740 | Yes |
| 80 | PKDCC |  |  | 243 | 0.741 | 0.4781 | Yes |
| 81 | EFNB1 |  |  | 244 | 0.740 | 0.4821 | Yes |
| 82 | PLAC9 |  |  | 254 | 0.731 | 0.4833 | Yes |
| 83 | ECM2 |  |  | 257 | 0.727 | 0.4867 | Yes |
| 84 | COL5A3 |  |  | 258 | 0.727 | 0.4906 | Yes |
| 85 | SCPEP1 |  |  | 259 | 0.727 | 0.4946 | Yes |
| 86 | PRKG1 |  |  | 261 | 0.726 | 0.4982 | Yes |
| 87 | IGFBP4 |  |  | 262 | 0.725 | 0.5022 | Yes |
| 88 | TWIST1 |  |  | 266 | 0.719 | 0.5052 | Yes |
| 89 | ATP2B1 |  |  | 270 | 0.713 | 0.5081 | Yes |
| 90 | ODF2L |  |  | 273 | 0.709 | 0.5114 | Yes |
| 91 | DAB2 |  |  | 277 | 0.702 | 0.5143 | Yes |
| 92 | FOXS1 |  |  | 282 | 0.698 | 0.5169 | Yes |
| 93 | SOCS1 |  |  | 292 | 0.686 | 0.5179 | Yes |
| 94 | CLMP |  |  | 307 | 0.665 | 0.5172 | Yes |
| 95 | TENM2 |  |  | 309 | 0.664 | 0.5205 | Yes |
| 96 | MYOF |  |  | 310 | 0.663 | 0.5242 | Yes |
| 97 | GBP3 |  |  | 316 | 0.658 | 0.5262 | Yes |
| 98 | PODNL1 |  |  | 331 | 0.644 | 0.5255 | Yes |
| 99 | COL18A1 |  |  | 336 | 0.638 | 0.5277 | Yes |
| 100 | TIAM1 |  |  | 341 | 0.636 | 0.5300 | Yes |
| 101 | PALLD |  |  | 342 | 0.636 | 0.5334 | Yes |
| 102 | TSHZ2 |  |  | 343 | 0.635 | 0.5369 | Yes |
| 103 | TRIM22 |  |  | 348 | 0.630 | 0.5391 | Yes |
| 104 | PROCR |  |  | 355 | 0.627 | 0.5407 | Yes |
| 105 | MAP1A |  |  | 358 | 0.626 | 0.5435 | Yes |
| 106 | CREB3L1 |  |  | 381 | 0.608 | 0.5401 | No |
| 107 | MDFIC |  |  | 389 | 0.601 | 0.5412 | No |
| 108 | ADAMTS12 |  |  | 404 | 0.582 | 0.5401 | No |
| 109 | GLI1 |  |  | 452 | 0.547 | 0.5288 | No |
| 110 | YIPF5 |  |  | 458 | 0.544 | 0.5302 | No |
| 111 | ABCA5 |  |  | 465 | 0.538 | 0.5313 | No |
| 112 | EDNRA |  |  | 479 | 0.529 | 0.5303 | No |
| 113 | CDC42EP5 |  |  | 484 | 0.524 | 0.5319 | No |
| 114 | TMEM263 |  |  | 502 | 0.509 | 0.5295 | No |
| 115 | LAMA2 |  |  | 506 | 0.503 | 0.5313 | No |
| 116 | ARHGAP29 |  |  | 508 | 0.502 | 0.5337 | No |
| 117 | TMEM98 |  |  | 510 | 0.502 | 0.5362 | No |
| 118 | PTCH2 |  |  | 541 | 0.486 | 0.5297 | No |
| 119 | PYCR1 |  |  | 563 | 0.474 | 0.5259 | No |
| 120 | DTNA |  |  | 587 | 0.460 | 0.5214 | No |
| 121 | LOXL2 |  |  | 589 | 0.459 | 0.5236 | No |
| 122 | TBC1D2B |  |  | 595 | 0.455 | 0.5245 | No |
| 123 | PDE5A |  |  | 608 | 0.447 | 0.5233 | No |
| 124 | THBS1 |  |  | 613 | 0.446 | 0.5245 | No |
| 125 | NUCB2 |  |  | 637 | 0.439 | 0.5199 | No |
| 126 | COPZ2 |  |  | 650 | 0.432 | 0.5186 | No |
| 127 | BST2 |  |  | 677 | 0.421 | 0.5130 | No |
| 128 | CPE |  |  | 680 | 0.420 | 0.5146 | No |
| 129 | ID1 |  |  | 682 | 0.420 | 0.5166 | No |
| 130 | SPATS2L |  |  | 710 | 0.408 | 0.5106 | No |
| 131 | PLIN3 |  |  | 714 | 0.406 | 0.5119 | No |
| 132 | AFDN |  |  | 738 | 0.401 | 0.5071 | No |
| 133 | NR2F2 |  |  | 741 | 0.400 | 0.5087 | No |
| 134 | DUSP1 |  |  | 787 | 0.386 | 0.4971 | No |
| 135 | NTN4 |  |  | 801 | 0.382 | 0.4952 | No |
| 136 | APOL1 |  |  | 811 | 0.378 | 0.4945 | No |
| 137 | ARL1 |  |  | 813 | 0.377 | 0.4963 | No |
| 138 | CYTOR |  |  | 835 | 0.370 | 0.4919 | No |
| 139 | MRC2 |  |  | 863 | 0.361 | 0.4856 | No |
| 140 | BNC2 |  |  | 864 | 0.361 | 0.4876 | No |
| 141 | ITGA5 |  |  | 874 | 0.357 | 0.4868 | No |
| 142 | CHN1 |  |  | 880 | 0.355 | 0.4872 | No |
| 143 | HTRA3 |  |  | 884 | 0.354 | 0.4882 | No |
| 144 | ITGA11 |  |  | 885 | 0.353 | 0.4902 | No |
| 145 | GUCY1B1 |  |  | 887 | 0.353 | 0.4918 | No |
| 146 | MIR22HG |  |  | 894 | 0.352 | 0.4919 | No |
| 147 | SAV1 |  |  | 905 | 0.350 | 0.4907 | No |
| 148 | SLIT3 |  |  | 911 | 0.348 | 0.4911 | No |
| 149 | CNPY4 |  |  | 912 | 0.348 | 0.4930 | No |
| 150 | IFI6 |  |  | 923 | 0.344 | 0.4918 | No |
| 151 | IFT20 |  |  | 929 | 0.343 | 0.4922 | No |
| 152 | LY6E |  |  | 1030 | 0.321 | 0.4635 | No |
| 153 | IGFBP6 |  |  | 1048 | 0.318 | 0.4600 | No |
| 154 | CGNL1 |  |  | 1099 | 0.307 | 0.4465 | No |
| 155 | WFDC1 |  |  | 1100 | 0.307 | 0.4482 | No |
| 156 | S1PR2 |  |  | 1104 | 0.305 | 0.4489 | No |
| 157 | PLAGL1 |  |  | 1132 | 0.301 | 0.4423 | No |
| 158 | GAREM1 |  |  | 1159 | 0.297 | 0.4360 | No |
| 159 | NR3C1 |  |  | 1162 | 0.297 | 0.4370 | No |
| 160 | EPB41L2 |  |  | 1170 | 0.295 | 0.4365 | No |
| 161 | TMEM119 |  |  | 1193 | 0.289 | 0.4314 | No |
| 162 | TSHZ3 |  |  | 1218 | 0.285 | 0.4256 | No |
| 163 | SIPA1L1 |  |  | 1254 | 0.276 | 0.4165 | No |
| 164 | CPED1 |  |  | 1309 | 0.267 | 0.4015 | No |
| 165 | ACTN1 |  |  | 1364 | 0.258 | 0.3865 | No |
| 166 | NUMBL |  |  | 1381 | 0.255 | 0.3830 | No |
| 167 | GLI2 |  |  | 1401 | 0.252 | 0.3786 | No |
| 168 | LRRN4CL |  |  | 1403 | 0.252 | 0.3796 | No |
| 169 | FARP1 |  |  | 1496 | -0.257 | 0.3530 | No |
| 170 | RAMP2 |  |  | 1566 | -0.263 | 0.3334 | No |
| 171 | CDKN1C |  |  | 1571 | -0.264 | 0.3337 | No |
| 172 | MAPK8IP1 |  |  | 1580 | -0.265 | 0.3327 | No |
| 173 | HIC1 |  |  | 1625 | -0.270 | 0.3207 | No |
| 174 | ERRFI1 |  |  | 1640 | -0.273 | 0.3180 | No |
| 175 | VEGFA |  |  | 1644 | -0.273 | 0.3185 | No |
| 176 | STARD13 |  |  | 1672 | -0.276 | 0.3118 | No |
| 177 | SCARA3 |  |  | 1701 | -0.280 | 0.3048 | No |
| 178 | GPR162 |  |  | 1709 | -0.281 | 0.3042 | No |
| 179 | TRAC |  |  | 1720 | -0.283 | 0.3027 | No |
| 180 | TGIF1 |  |  | 1955 | -0.313 | 0.2332 | No |
| 181 | TLE2 |  |  | 1981 | -0.316 | 0.2273 | No |
| 182 | ANGPTL1 |  |  | 2021 | -0.321 | 0.2172 | No |
| 183 | CDKN1A |  |  | 2083 | -0.332 | 0.2004 | No |
| 184 | ADAM22 |  |  | 2159 | -0.344 | 0.1795 | No |
| 185 | TRIL |  |  | 2250 | -0.363 | 0.1540 | No |
| 186 | STIM2 |  |  | 2256 | -0.364 | 0.1545 | No |
| 187 | ETV4 |  |  | 2325 | -0.378 | 0.1358 | No |
| 188 | CTTNBP2 |  |  | 2385 | -0.390 | 0.1200 | No |
| 189 | CTDSPL |  |  | 2419 | -0.397 | 0.1121 | No |
| 190 | KCTD9 |  |  | 2438 | -0.399 | 0.1088 | No |
| 191 | TLE1 |  |  | 2502 | -0.415 | 0.0919 | No |
| 192 | PLPPR5 |  |  | 2521 | -0.419 | 0.0887 | No |
| 193 | SSBP4 |  |  | 2596 | -0.438 | 0.0686 | No |
| 194 | PLK2 |  |  | 2720 | -0.476 | 0.0337 | No |
| 195 | TMEM59L |  |  | 2777 | -0.496 | 0.0194 | No |
| 196 | JAM2 |  |  | 2784 | -0.498 | 0.0202 | No |
| 197 | PLXDC1 |  |  | 2835 | -0.514 | 0.0078 | No |
| 198 | CAPN5 |  |  | 2880 | -0.531 | -0.0027 | No |
| 199 | TMEM158 |  |  | 2900 | -0.543 | -0.0055 | No |
| 200 | SHROOM3 |  |  | 2905 | -0.545 | -0.0038 | No |
| 201 | MRAS |  |  | 2912 | -0.547 | -0.0026 | No |
| 202 | SPRY4 |  |  | 2916 | -0.552 | -0.0005 | No |
| 203 | AC009041.2 |  |  | 2963 | -0.580 | -0.0114 | No |
| 204 | KLHL13 |  |  | 2965 | -0.580 | -0.0085 | No |
| 205 | ITPR1 |  |  | 3029 | -0.619 | -0.0243 | No |
| 206 | RGS16 |  |  | 3134 | -0.708 | -0.0521 | No |
| 207 | SPRY1 |  |  | 3163 | -0.746 | -0.0566 | No |
| 208 | TCF4 |  |  | 3205 | -0.787 | -0.0648 | No |
| 209 | GAD1 |  |  | 3211 | -0.796 | -0.0620 | No |
| 210 | SULF2 |  |  | 3233 | -0.823 | -0.0639 | No |
| 211 | C16orf45 |  |  | 3250 | -0.850 | -0.0641 | No |
| 212 | SOX9 |  |  | 3271 | -0.883 | -0.0654 | No |
| 213 | ZFHX4 |  |  | 3291 | -0.915 | -0.0662 | No |
| 214 | FRMD4B |  |  | 3294 | -0.917 | -0.0618 | No |
| 215 | MEST |  |  | 3307 | -0.940 | -0.0604 | No |
| 216 | PLPP3 |  |  | 3335 | -0.988 | -0.0632 | No |
| 217 | CDK6 |  |  | 3366 | -1.085 | -0.0664 | No |
| 218 | ADAMTS9 |  |  | 3407 | -1.260 | -0.0718 | No |
| 219 | RASSF2 |  |  | 3421 | -1.303 | -0.0686 | No |
| 220 | CLU |  |  | 3424 | -1.329 | -0.0620 | No |
| 221 | IGFBP3 |  |  | 3433 | -1.384 | -0.0569 | No |
| 222 | CXCL14 |  |  | 3461 | -1.710 | -0.0558 | No |
| 223 | SCRG1 |  |  | 3480 | -1.876 | -0.0511 | No |
| 224 | NGFR |  |  | 3494 | -2.256 | -0.0428 | No |
| 225 | NRP2 |  |  | 3502 | -2.549 | -0.0310 | No |
| 226 | DCX |  |  | 3505 | -2.772 | -0.0165 | No |
| 227 | PDGFRA |  |  | 3508 | -3.318 | 0.0009 | No |
Table: GSEA details [plain text format]

  

Fig 2: DOROSZKO\_U3054MG\_CLUSTER\_2: Random ES distribution      
 Gene set null distribution of ES for **DOROSZKO\_U3054MG\_CLUSTER\_2**
